# Characterization of the Principal Constituents of Danning Tablets, a Chinese Formula Consisting of Seven Herbs, by an UPLC-DAD-MS/MS Approach

**DOI:** 10.3390/molecules21050631

**Published:** 2016-05-14

**Authors:** Changsen Zhan, Aizhen Xiong, Danping Shen, Li Yang, Zhengtao Wang

**Affiliations:** 1 The MOE Key Laboratory for Standardization of Chinese Medicines and the SATCM Key Laboratory for New Resources and Quality Evaluation of Chinese Medicines, Institute of Chinese Materia Medica, Shanghai University of Traditional Chinese Medicine, Shanghai 201203, China; zhanchangsen@shpl.com.cn (C.Z.); a.z.xiong@hotmail.com (A.X.); 2 Shanghai Hutchison Pharmaceuticals Co., Ltd., Shanghai 200331, China; dpshen@shpl.com.cn; 3 Shanghai Engineering Research Center for Innovation of Solid Preparation of Traditional Chinese Medicine, Shanghai 200331, China; 4 Shanghai R & D Center for Standardization of Traditional Chinese Medicines, Shanghai 201203, China

**Keywords:** Danning Tablets, *Rhei Radix et Rhizoma*, *Polygoni Cuspidati Rhizoma et Radix*, anthraquinones, UPLC-DAD-ESI-MS/MS, traditional Chinese medicine

## Abstract

Danning Tablets are a traditional Chinese formula showing broad clinical applications in hepatobiliary diseases and containing a diversity of bioactive chemicals. However, the chemical profiling of the formula, which serves as the material foundation of its efficacy, is really a big challenge as Danning Tablets consist of seven herbs from different origins. An ultra-performance liquid chromatography coupled to diode array detection and electrospray ionization mass spectrometry (UPLC-DAD-ESI-MS/MS) approach was developed to characterize the principal polyphenol constituents in the formula. As a result, a total of 32 constituents, including 14 anthraquinones and their glucosides, four anthrones, two naphthalene glycosides, two stilbenes and 10 flavonoids were identified based on their retention time, UV absorption and MS/MS fragmentation patterns. The sources of these compounds were also illustrated. Most of the bioactive anthraquinone derivatives were found in *Rhei Radix et Rhizoma* or *Polygoni Cuspidati Rhizoma et Radix*, which are the Emperor drugs in the formula for its clinic usage. These findings indicate the merit of using this integrated UPLC-DAD-ESI-MS/MS approach to rapidly illustrate the chemical foundation of complex formulas. The present study will facilitate the quality control of Danning Tablet formulas as well as the individual herbs.

## 1. Introduction

Herbal medicines have been used over thousands of years as alternative medicines or health products in China and many other countries, such as Korea, Japan, India, as well as in Western societies. Nowadays it well recognized that the biological activities of herbal medicines are the sum contribution of the polyvalent effects of their co-existing components. Therefore, great attention should be paid to the quality assessment of herbal medicines and formulas in order to guarantee their efficacy and safety.

Danning Tablets are a traditional Chinese formula showing a broad range of clinical applications in hepatobiliary diseases such as chronic cholecystitis, gallstones, constipation, and non-alcoholic fatty liver [1,2,3]. It is proven to have anti-inflammatory, and anti-stone properties, and to improve hepatic steatosis thus protecting the liver and gall bladder from injuries [4,5,6]. It is officially recorded in the Chinese Pharmacopoeia and consists of seven herbs according to traditional Chinese medicine formulation theory, *i.e.*, *Rhei Radix et Rhizoma* (RRR), *Polygoni Cuspidati Rhizoma et Radix* (PCRR), *Citri Reticulatae Pericarpium* (CRP), *Citri Reticulatae Pericarpium Viride* (CRPV), *Curcumae Radix* (CR), *Crataegi Fructus* (CF) and *Imperatae Rhizoma* (IR). In the formula, RRR and PCRR are regarded as the Emperor (Jun) drugs, which means that RRR and PCRR are the major herbs responsible for the therapeutic effects of Danning Tablet in liver and gall bladder diseases. As reported, various components with pharmacological actions have been found in the formula, mainly anthraquinone derivatives such as emodin, chrysophanol, rhein, aloe-emodin, physcion and their glucosides [7,8]. Besides, flavonoids and stilbenes are also found in the formula. These two kinds of compounds are dose-dependently responsible for the protective effect of the formula on ANIT-induced liver injury with cholestasis in rats, which is related to the attenuation of oxidative stress in the liver tissue and neutrophil infiltration [9].

Danning Tablet formulations are prepared from seven medicinal herbs from different origins, each of which contains a diverse chemical profile. Therefore, the complete chemical profiling of the formulae is a rather complex problem, and it is undoubtedly a big challenge to identify the chemical markers with biological activity in such a highly complicated system. However, the knowledge about the chemicals in the formula is urgently needed for: (1) better understanding of its pharmacological mechanism(s) of action and (2) quality control of the formula via monitoring the multiple components. Therefore, great attention should be paid on characterizing the bioactive constituents in the formula.

Traditional quality control methods are mainly based on quantifying a single marker substance. Recently, chromatographic fingerprints have shown great advantage over traditional methods in mapping the entire metabolic profile of herbal medicines. In the last decade, liquid chromatography coupled with mass spectrometry (LC-MS) has become a powerful technique for the analysis of complex botanical extracts [10]. However, ultra-high performance liquid chromatography coupled to diode array detection and mass spectrophotometer (UPLC-DAD-MS) has recently been widely used in this area because it can achieve both higher sensitivity and shorter analysis times [11], which is essential for efficient analysis of trace amounts of chemicals. UPLC uses high pressure pumps to accommodate the use of sub-2 μm particle size columns, which can contribute to robust and rapid sample analysis [12]. DAD provides abundant information for structural elucidation of the compounds when it is integrated with tandem mass spectrometry. The electrospray ionization (ESI) source is preferred for its high ionization efficiency for the majority of chemical structures, such as phenolic compounds [13]. Hence, the combined technique of UPLC-DAD-ESI-MS/MS facilitates rapid and accurate identification of chemical compounds in complex Chinese medicines and formulas.

A chemical fingerprint and a simultaneous analysis of eight bioactive compounds in Danning Tablets were reported [14,15], which were inadequate to clearly reflect the intrinsic properties of such a complex formula containing seven botanical drugs. Besides, the bioactive constituents of Danning Tablets are still not clear. In the present study, we aimed to identify the structures and sources of polyphenols in the formula, including anthraquinones, anthrones, naphthalene, stilbenes and flavonoids. It was found that among all the 32 chemicals identified in the formula, 24 compounds were generated from RRR or PCRR, which are the major drugs responsible for the therapeutic effects of this formula. The anthraquinones profile, *i.e.*, free anthraquinones, anthraquinone glycosides, anthrones and bianthrones, may serve as a diagnostic chemical marker for the quality assessment of Danning Tablets and provide information for a better understanding of the biological activities.

## 2. Results and Discussion

### 2.1. Optimization of the UPLC and MS Conditions

Danning Tablet is specially formulated using seven kinds of herbs from different origins. A vast number of chemical components co-exist in the formula, which makes the identification of the bioactive components more challenging. Therefore, the analysis conditions should be optimized to obtain chromatograms with good separation as well as sensitivity.

**Figure 1:**
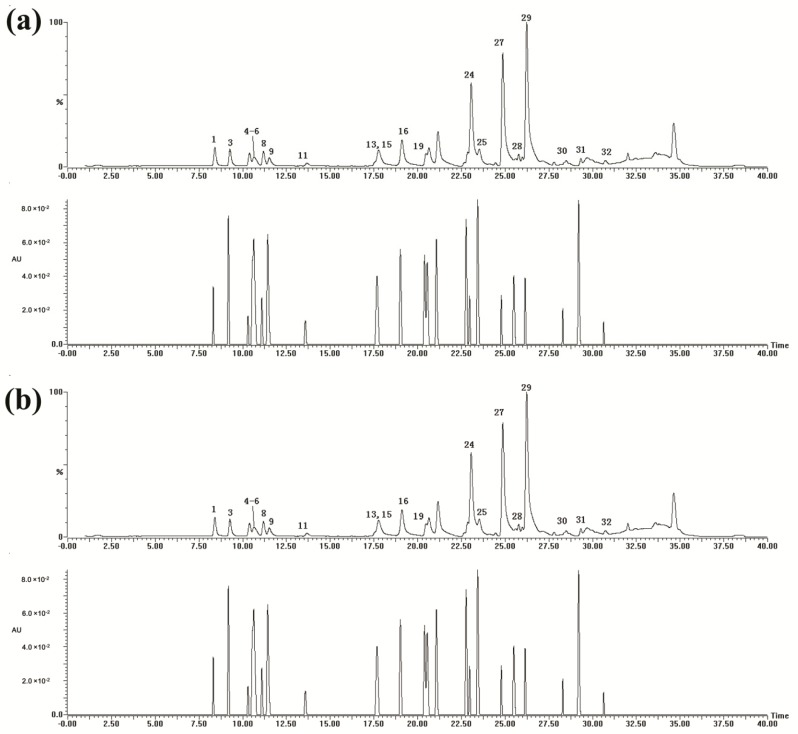
UPLC-DAD-ESI-MS chromatograms of standard reference mixture (**a**) and Danning Tablets sample (**b**) (Upper panel, LC-MS chromatogram at positive ion mode; lower panel, LC-UV chromatogram at 266 nm. Peaks 1–32 were same as those in Table 1).

Firstly, parameters including column types, column temperatures, mobile phase, elution program, flow rates and detection wavelengths were investigated. Among the columns tested, T3 column (2.1 mm × 100 mm, 1.8 μm, Waters, Milford, MA, USA) was proved to be better than another two, *i.e.*, a C8 column (2.1 mm × 100 mm, 1.8 μm, Waters) and an ODS column (2.0 mm × 75 mm, 1.6 μm, Shimadzu, Nakagyo, Kyoto, Japan). The best separation was achieved when the column temperature was set at 35 °C other than 30 and 40 °C. Formic acid (0.1%) was used to improve the chromatographic behavior and to reduce the peak tailing. Five wavelengths were tested, *i.e.*, 210 nm, 254 nm, 266 nm, 280 nm and 346 nm. And the best separation with most abundant peak number and highest intensities was achieved at 266 nm (Figure 1). Besides, better separation was obtained when the flow rate was lower, *i.e.*, at 0.2 mL/min.

For ESI-MS detection, the positive ion mode was selected for its better sensitivity and separation than the negative ion mode. As results, the condition was optimized as described in the Experimental Section. The LC-DAD-MS chromatogram of 20 reference standards is shown in Figure 1a, which shows clearly the separation of all standard references. A typical chromatogram of a Danning Tablets sample is shown in Figure 1b.

### 2.2. UPLC-DAD-ESI-MS/MS Analysis of Compounds in Danning Tablets

A total of 32 compounds were identified by interpretation of their t_R_ value, UV absorbance, molecular weight, and characteristic MS/MS fragment ions. All the compounds could be classified into five groups: anthraquinones, anthrones, naphthalenes, stilbenes and flavonoids. Twenty compounds were unambiguously confirmed by comparison with their standard references (Table 1).

**Figure 2:**
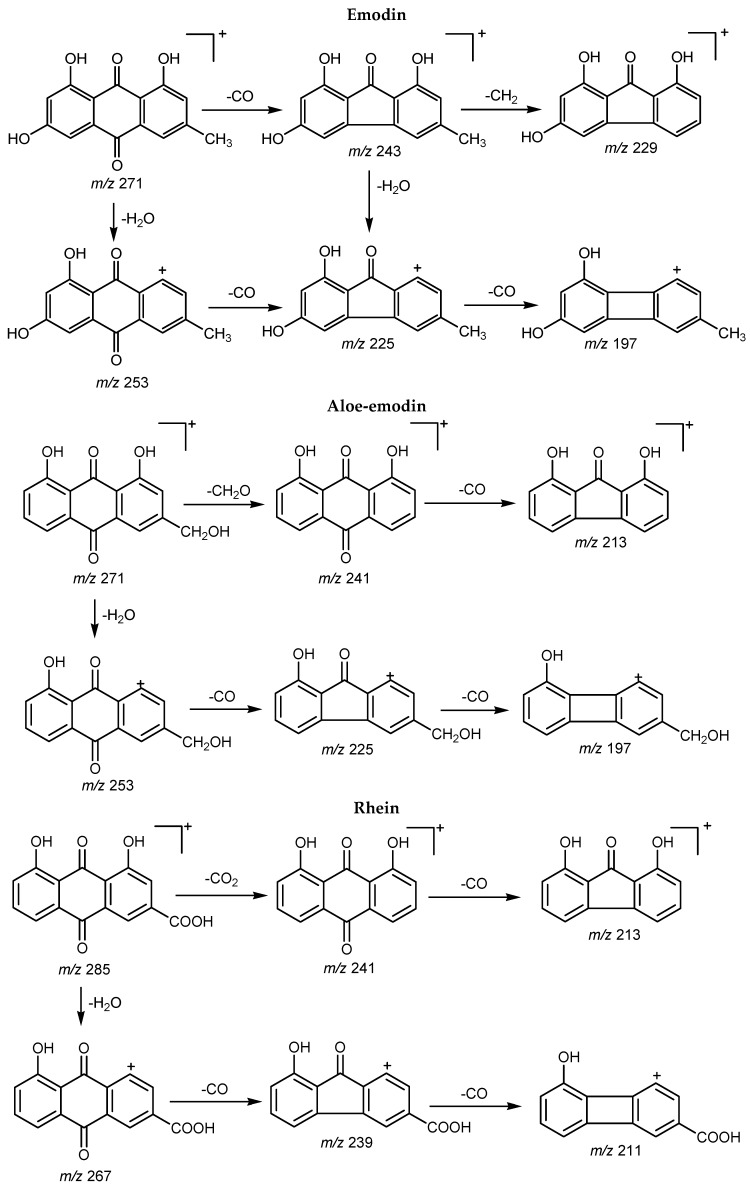

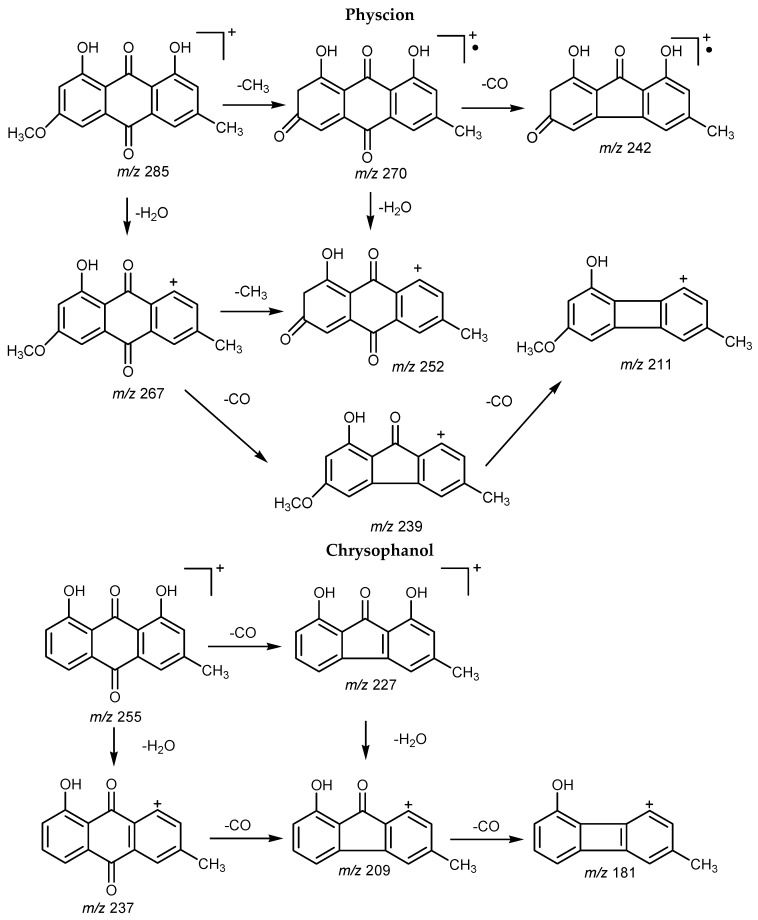
Proposed MS/MS fragmentation pathways of five free anthraquinones standard references.

Standard references for five anthraquinones and one glycoside, *i.e.*, aloe-emodin, rhein, emodin, chrysophanol, physcion, and emodin 8-*O*-β-d-glucoside, were used in the UPLC-DAD-MS/MS analysis to obtain general information about the free anthraquinones and their glycosides (Figure 2 and Figure S1 in the Supplementary Materials). A great similarity was found in their UV absorption and MS/MS fragmentation. Firstly, both the free anthraquinones and the glycosides exhibited characteristic maximum absorptions at 250–270, 280–290 and 425–440 nm. The glycosides firstly lost 162 Da, *i.e.*, elimination of a glucosyl residue, to produce a predominant fragment with an *m*/*z* equal to its corresponding free anthraquinone, which could be obviously observed even in their full scan MS spectra (Figure S2). Then, a series of losses of 18 and 28 Da was typical for free anthraquinones containing hydroxyl (OH) and carbonyl (CO) groups on the benzene ring [16]. For example, emodin easily produced a *m*/*z* 243 peak by the elimination of CO, which was then followed by loss of a CH_2_ group to give *m*/*z* 229. In the MS/MS spectrum of chrysophanol, a product ion at *m*/*z* 227 was observed, which resulted from the direct loss of CO from [M + H]^+^. Both the *m*/*z* 229 fragment for emodin and the *m*/*z* 227 one of chrysophanol were very stable and did not yield any further fragmentations. Because the C-9 carbonyl group has intramolecular hydrogen bonding with the C-1 and C-8 α-hydroxyl groups it is difficult to cleave, therefore, the CO elimination possibly originated from the C-10 carbonyl group. The glucosyl residue of emodin 8-*O*-β-d-glucoside was lost very easily to produce a moiety which was then fragmented in the same pattern as that of emodin. Free anthraquinones containing other functional groups on the benzene ring, such as COOH, CH_2_OH, COH_3_, lost these groups easily to produce the corresponding fragments. As seen in the MS/MS fragmentation of aloe-emodin, physcion and rhein, fragments of *m*/*z* 241, *m*/*z* 270 and *m*/*z* 241 were produced by the losses of CH_2_O, CH_3_, and CO_2_, respectively (Figure 2 and Figure S1). Besides, these differences were also valuable for the identification of isomer pairs, such as emodin and aloe-emodin, physcion and rhein, as well as of their corresponding glycosides. Therefore, five free anthraquinones (peaks 25, 28, 30, 31 and 32) were identified as aloe-emodin, rhein, emodin, chrysophanol and physcion, respectively, by comparing their retention times, UV absorptions, and MS/MS fragmentation patterns with those of the standard references and literature data [16,17,18]. Besides, nine anthraquinone glycosides (peaks 2, 7, 10, 12, 17, 18, 19, 21 and 23) were identified (Figure S3). Among them, peak 2 (t_R_ 9.40 min) and 10 (t_R_ 12.14 min) were tentatively characterized as aloe-emodin-*O*-glucoside isomers, showing characteristic MS/MS fragment ions at *m*/*z* 271, 253, 241, 225, 213 and 197, which was similar to the MS/MS fragmentation behavior of aloe-emodin, *i.e.*, *m*/*z* 253, 241, 225, 213 and 197. However, the identification of the glucosylation position was not possible as too many possible positions exist [16]. Peak 7 (t_R_ 11.10 min) was fragmented to produce a peak with *m*/*z* 285, which was further fragmented in a similar pattern as that of rhein, *i.e.*, producing ions at *m*/*z* 267, 241, 239, 213 211 and 185. Thus, peak 7 was tentatively identified as rhein-*O*-glucoside but without specifying the glucosyl position because at least two possible positions were reported [16]. Similarly, peaks 19 (t_R_ 20.53 min) and 12 (t_R_ 16.00 min) were identified as emodin-8-*O*-β-d-glucoside and its isomer, respectively. Peaks 17 (t_R_ 19.60 min) and 18 (t_R_ 19.95 min) were tentatively characterized as chrysophanol-*O*-glucoside isomers [16]. Meanwhile, peak 21 (t_R_ 22.02 min) and 23 (t_R_ 22.71 min) were tentatively identified as physcion 1-*O*-glucoside and physcion 8-*O*-glucoside, respectively (Figure S2).

Four glycosyl anthrone standards, *i.e.*, sennoside A, sennoside B, sennoside C, and aloin, were applied in the UPLC-DAD-MS/MS analysis to get general information about these components (Figure S1). The characteristic UV maximum absorptions for these compounds were found at 260–275 and 320–360 nm, which facilitated their preliminary identification (Table 1). All three bianthrones, *i.e.*, sennoisde A, B and C, were positively ionized to lose two glycosyl groups and cleaved at the C10–C10′ bond to produce a significant ion at *m*/*z* 270, which was relatively hard to further fragment and thus could be used for rapid identification of anthrones in the present study. Although being similar in structure, these three diglycosyl anthrones showed different retention behavior on the column and peaks 4 (t_R_ 10.92 min), 9 (t_R_ 11.73 min), and 11 (t_R_ 14.07 min), were identified as sennoside B, sennoside C and sennoside A [16]. In addition to the maximum UV absorptions at 269 and 358 nm, aloin also showed a specific absorption at 296 nm. It was fragmented to lose one glycosyl group and produced an ion at *m*/*z* 257, which further lost H_2_O and CO to produce ions at *m*/*z* 239 and 211, respectively. Therefore, peak 15 (t_R_ 18.11 min) was identified as aloin [19].

Two peaks, *i.e.*, peak 14 (t_R_ 18.14 min) and peak 20 (t_R_ 20.92 min), exhibited maximum UV absorptions at 246, 265 and 323 nm. Besides, both of them gave the same fragment ions at *m*/*z* 409, 431 and 247, suggesting common base peaks at *m*/*z* 409 ([M + H]^+^) and the existence of a glycosyl group (Table 1). The ion at *m*/*z* 247 further fragmented to produce ions at *m*/*z* 229, 214 and 201, which were caused by successive losses of H_2_O and CO from the *m*/*z* 247 ion, and loss of CO from the ion at *m*/*z* 229. Therefore, peaks 14 and 20 were tentatively identified as torachrysone-*O*-glucoside or its isomer according to the reported data [20].

Two stilbenes standards, *i.e.*, piceid and resveratrol, were used for the UPLC-DAD-MS/MS analysis (Figure S4). Characteristic maximum UV absorptions were found at 230 and 305 nm (Table 1). Piceid gave a [M + H]^+^ ion at *m*/*z* 391 and a product ion of *m*/*z* 229 by elimination of a glucosyl residue. Resveratrol, the aglycon of piceid, gave a [M + H]^+^ ion at *m*/*z* 229 and produced fragments at *m*/*z* 211, 183, 165, 135 and 119. The ions at *m*/*z* 211 and 183 were produced by successive losses of H_2_O and CO from the parent ion. Meanwhile, the ions at *m*/*z* 135 and 119 correspond to the cleavage of the A–B and C–D bonds of the parent ion at *m*/*z* 229. Besides, the ion at *m*/*z* 165 was caused by loss of H_2_O from the ion at *m*/*z* 183. Therefore, peaks 1 (t_R_ 8.52 min) and 8 (t_R_ 11.46 min) were identified as piceid and resveratrol, respectively, by comparing their retention times, UV absorption, MS and MS/MS fragmentation patterns with those of the reference compounds. No other stilbenes were detected in the formula.

In total, ten flavonoids were identified in Danning Tablets, including seven flavone structures, one flavanone structure and two flavonol structures (Figure S5). All of these compounds exhibited two maximum UV absorptions at 240–285 and 300–400 nm originating from their A- and B-rings, which are typical for flavonoids. Eight standard references were investigated to get the characteristic fragmentations for each subtype. Generally, loss of 28 Da (CO) and 44 Da (CO_2_) from the C–ring was common for all the standard references investigated, while loss of 15 Da (CH_3_) was specific for compounds those contain a methoxyl group. Besides, the structures with different aglycones, *i.e.*, methoxyl substitution, hydroxyl substitution, *C*-glycosyl groups and *O*-glycosyl groups, showed different MS/MS fragmentation patterns [21,22,23,24,25,26]. As a result, peaks 3 (t_R_ 9.42 min), 5 (t_R_ 11.02 min), 6 (t_R_ 11.03 min), 13 (t_R_ 18.12 min), 16 (t_R_ 19.54 min), 24 (t_R_ 23.19 min), 27 (t_R_ 25.02 min) and 29 (t_R_ 26.35 min) were unequivocally identified as vitexin, hyperoside, hesperidin, quercetin, luteolin, sinensetin, nobiletin and tangeretin, respectively. Peak 22 (*m*/*z* 373 at t_R_ 22.62 min) showed a specific flavonoid UV absorption. Besides, peak 22 fragmented in a way similar to that of sinensetin, *i.e.* producing ions at *m*/*z* 343 and 329. Therefore, peak 22 was tentatively identified as isosinensetin according to [27,28]. Besides, peak 26 (*m*/*z* 343 at t_R_ 24.17 min) was tentatively identified as tetramethyl-*O*-isoscutellarein by comparing its MS/MS fragmentation to that of the reported data [28].

### 2.3. Confirmation of the Source of the Compounds Identified in the Formula

**Figure 3:**
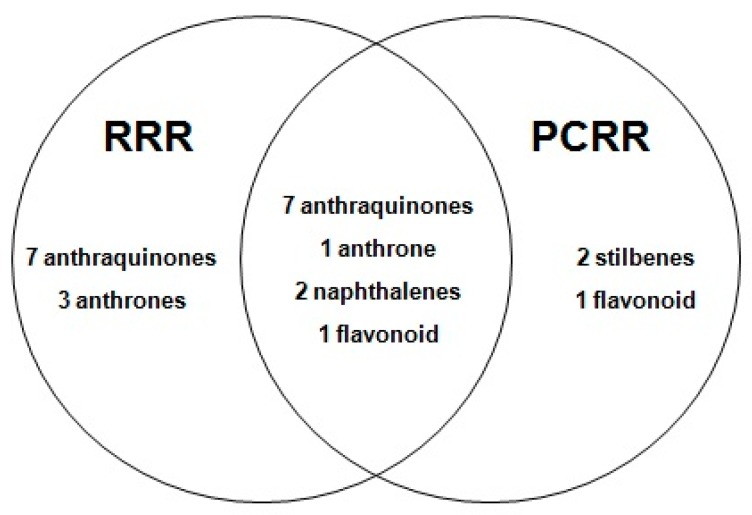
The compounds identified in *Rhei Radix et Rhizoma* (RRR) and *Polygoni Cuspidati Rhizoma et Radix* (PCRR).

Furthermore, the sources of these 32 compounds were identified by comparisons between the complete formula containing all seven herbs, the formulae without certain herbs and each of the single herbs (Table 1). For example, a comparison was firstly made between the complete formula and the single RRR herb, and 21 compounds were identified as coming from RRR (Figure S6). However, when a further comparison was made between the complete formula, the revised formula consisting of six herbs (without RRR, Figure S7), and the single PCRR herb, 11 compounds were also found to exist in RRR, therefore, only 10 chemicals were identified to be uniquely sourced from RRR, including aloe-emodin-*O*-glucoside, sennoside B, rhein-*O*-glucoside, sennoside C, sennoside A, chrysophanol 1-*O*-glucoside, chrysophanol 8-*O*-glucoside, rhein and chrysophanol. Similarly, 14 compounds were sourced from PCRR, among which only two chemicals, *i.e.*, piceid and resveratrol, were unique to PCRR. Nine compounds were sourced from CRPV, among which six (vitexin, isosinensetin, sinensetin, tetramethyl-*O*-isoscutellarein, nobiletin and tangeretin) were unique to CRPV. Three compounds were found in CF, among which two chemicals were uniquely found in CF, namely hyperoside and quercetin. What’s more, 24 compounds were generated from RRR or PCRR, including 14 anthraquinones, four anthrones, two stilbenes, two naphthalenes, and two flavonoids. Twelve were found to be co-existing compounds in RRR and PCRR (Figure 3). It’s known that RPP and PCRR are the Emperor (Jun) drugs in the Danning Tablet formula, indicating the importance of RPP and PCRR for its therapeutic effects. Therefore, the traditional formulation theory is consistent with the chemical foundation of the formula as revealed in our present study.

## 3. Experimental Section

### 3.1. Materials

HPLC-grade methanol and formic acid (96%) were obtained from Tedia Co. (Fairfield, OH, USA). Water was prepared by a Milli-Q system (Millipore, MA, USA). All other reagents were of analytical grade and were purchased from Tianjin Damao Chemical Reagent Factory (Tianjin, China). Twenty reference compounds (Table 2) were purchased from the National Institute for Food and Drug Control (Beijing, China). The herbs for preparation of Danning Tablets were provided by Shanghai Hutchison Pharmaceuticals (Shanghai, China). Danning Tablets are made with seven herbal drugs, *i.e*., *Rhei Radix et Rhizoma* (RRR), *Polygoni Cuspidati Rhizoma et Radix* (PCRR), *Citri* *Reticulatae Pericarpium* (CRP), *Citri Reticulatae Pericarpium Viride* (CRPV), *Curcumae Radix* (CR), *Crataegi Fructus* (CF) and *Imperatae Rhizoma* (IR), and some excipients. The amounts of each composition for 1000 tablets are as follows: RRR, 48 g; PCRR, 720 g; CRPV, 288 g; CRP, 288 g; CR, 432 g; CF, 720 g; IR, 432 g; calcium sulphate, 6.25%; d-(+)-sucrose, 7.5%; povidone K 30, 1.25%; magnesium stearate, 1%; sodium bicarbonate, 2%. Danning Tablets and all herbs were stored in the dark at room temperature and verified by the Quality Inspection Center of Shanghai Hutchison Pharmaceuticals. Qualified inspection reports were provided according to the Chinese Pharmacopoeia standards. The seven revised formulas of Danning Tablets with only six herbs were prepared according to the formulation of the complete formula but with exclusion of each single herb, respectively.

### 3.2. Sample Preparation

The Danning Tablets were firstly powdered and 1 g of the powder was extracted twice with 50 mL of 95% ethanol under ultrasound for 60 min. After filtering, the filtrates were combined and 95% ethanol was added to the volumetric flask up to 100 mL. A precisely measured aliquot (25 mL) was evaporated and methanol added up to 10 mL. The supernatant solution was filtrated through a syringe filter (0.22 μm) and aliquots (2 μL) were injected into the chromatographic system for analysis. About 0.5 g of the powdered single herb was accurately measured and prepared following the method described above for analysis.

### 3.3. Equipment and Chromatographic Conditions

A Waters Acquity system (Waters Corp., Milford, MA, USA) was coupled with a triple-quadrupole tandem mass spectrometry, a gradient pump, an autosampler and a DAD detector. Chromatographic separation was carried out on a Waters ACQUITY UPLC HSS T3 column (2.1 mm × 100 mm, 1.8 μm) at a column temperature of 35 °C, with a flow rate of 0.2 mL/min. The mobile phase were composed of methanol (**A**) and 0.1% formic acid (**B**) with a gradient program as follows: 0–3 min, 5%–16% **A**; 3–4.5 min, 16%–32% **A**; 4.5–6.5 min, 32%–40% **A**; 6.5–10 min, 40%–43% **A**; 10–15 min, 43% **A**; 15–17 min, 43%–52% **A**; 17–21.5 min, 52%–65% **A**; 21.5–28 min, 65%–85% **A**; 28–30 min, 85%–92% **A**; 30–33 min, 92%–95% **A**. A pre-equilibration period of 6 min was used between individual run. The DAD detector was set in the range of 200–500 nm. Mass spectrometry was performed in the ESI positive ion mode. Data acquisition was performed in full-scan, selective ions monitoring (SIR) and MS/MS modes in the range of *m*/*z* 150–1000. The capillary voltage was optimized to 4 kV, and the cone voltage was 30 V. The source temperature was set to 120 °C, and the desolvation temperature was 350 °C, while the desolvation gas flow was set to 600 L/h, and the cone gas flow was 100 L/h.

## 4. Conclusions

A total of 32 compounds, including five free anthraquinones, nine anthraquinone glucosides, three dianthrone glycosides, one anthrone glycoside, two naphthalene glycosides, two stilbenes, and 10 flavonoids, were successfully identified from the traditional Chinese medicine formula Danning Tablets by UPLC-DAD-ESI-MS/MS by comparison with reference standards and their UV absorption features and MS/MS fragmentation patterns. Meanwhile, the source of these 32 compounds was identified by comparison among Danning tablets, revised Danning tablets lacking a single component and the single herbs. It was found that among all the 32 chemicals identified in the formula, 24 originated from *Rhei Radix et Rhizoma* (RRR) or *Polygoni Cuspidati Rhizoma et Radix* (PCRR), among which there were 14 anthraquinones and four anthrones. As reported, anthraquinone derivatives are the main chemicals responsible for the activity of the Danning Tablet formula. It’s also known that RRR and PCRR are the major drugs responsible for the therapeutic effects of this formula. These two matched well, thus revealing the success of our proposed approach for illustrating the chemical foundation for the biochemical activities of traditional formulas.

The current developed method also achieved rapid identification of the chemical components in Danning Tablets thus providing detailed information for establishing a comprehensive quality control method. Furthermore, identification of the source of each component in Danning Tablets will also benefit the quality control of herbs, so as to achieve better quality control of Danning Tablets. In addition, it also provided a basis for profiling the active components of the formula in biological samples, such as serum, which would facilitate metabolism and pharmacokinetic studies of the formula to further illustrate the mechanism of action of the formula.

## Figures and Tables

**Table 1:** Chemicals characterized in Danning tablets by UPLC-DAD-ESI-MS/MS analysis.

Peak No.	t_R_ (min)	UV _λ_ (nm)	MW	[M + H]^+^	CE (eV)	MS/MS Fragmentation	Identification
*Stilbenes*							
1	8.52	230, 305	390	391	20	229	Piceid ^a^
8	11.46	230, 305	228	229	30	211, 183, 165, 135, 119	Resveratrol ^a^
*Anthraquinones*							
2	9.4	256, 280, 430	432	433	25	271, 253, 241, 225, 213, 197	Aloe-emodin-*O*-glucoside isomer
7	11.10	258, 280, 430	446	447	25	285, 267, 241, 239, 213, 211, 185	Rhein-*O*-glucoside
10	12.14	256, 280, 430	432	433	30	271, 253, 241, 225, 213, 197	Aloe-emodin-*O*-glucoside isomer
12	15.97	256, 280, 430	432	433	25	271, 253, 243, 229, 225, 197	Emodin-*O*-glucoside
17	19.60	256, 285, 430	416	417	25	255, 237, 227, 209, 181	Chrysophanol 1-*O*-glucoside
18	19.97	256, 285, 430	416	417	25	255, 237, 227, 209, 181	Chrysophanol 8-*O*-glucoside
19	20.53	256, 282, 430	432	433	25	271, 253, 243, 229, 225, 197	Emodin 8-*O*-β-d-glucoside ^a^
21	22.02	256, 284, 430	446	447	30	285, 270, 267, 252, 242, 239, 211	Physcion 1-*O*-glucoside
23	22.71	256, 284, 430	446	447	30	285, 270, 267, 252, 242, 239, 211	Physcion 8-*O*-glucoside
25	23.71	256, 280, 430	270	271	27	253, 241, 225, 213, 197	Aloe-emodin ^a^
28	25.52	258, 280, 431	284	285	25	267, 241, 239, 213, 211, 185	Rhein ^a^
30	28.52	265, 287, 437	270	271	27	253, 243,229, 225, 197	Emodin ^a^
31	29.41	257, 287, 429	254	255	25	237, 227, 209, 181	Chrysophanol ^a^
32	30.78	265, 284, 434	284	285	27	270, 267, 252, 242, 239, 211	Physcion ^a^
*Anthrones*							
4	10.92	267, 357	863	270 ^b^	20	270	Sennoside B ^a^
9	11.73	268, 322	849	256 ^b^	20	256	Sennoside C ^a^
11	14.07	268, 341	863	270 ^b^	20	270	Sennoside A ^a^
15	18.16	269, 296, 358	418	419	20	257, 239, 211	Aloin ^a^
*Naphthalenes*							
14	18.14	246, 265, 339	408	409	25	247, 229, 214, 201	Torachrysone-*O*-glucoside isomer
20	20.92	246, 265, 339	408	409	25	247, 229, 214, 201	Torachrysone-*O*-glucoside isomer
*Flavonoids*							
3	9.42	268, 341	432	433	20	313, 283	Vitexin ^a^
5	11.02	255, 355	464	465	20	303	Hyperoside ^a^
6	11.03	231, 284	610	611	15	449, 303	Hesperidin ^a^
13	18.12	255, 371	302	303	25	285, 275, 257, 247, 229, 219, 201	Quercetin ^a^
16	19.54	253, 350	286	287	30	269, 241, 179, 161, 153, 135	Luteolin ^a^
22	22.62	271, 340	372	373	25	358, 343, 329	Isosinensetin
24	23.19	288, 339	372	373	25	358, 343, 329, 312, 297	Sinensetin ^a^
26	24.17	270, 335	342	343	25	328, 313, 285	Tetramethyl-*O*-isoscutellarein
27	25.02	271, 336	402	403	25	388, 373, 355, 342, 327	Nobiletin ^a^
29	26.35	272, 326	372	373	25	358, 343, 312, 297	Tangeretin ^a^

^a^ Means the chemical was authorized by standard reference; ^b^ Means the *m*/*z* was responsible for [M + H − 2Glu − C_15_H_9_O_5_]^+^; CE means collision energy used for MS/MS fragmentation.

**Table:** **(a) Free anthraquinones and their glucosides** 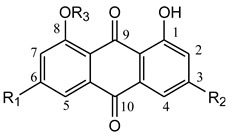

No.	Compound Name	R_1_	R_2_	R_3_
1	Chrysophanol	H	CH_3_	H
2	Emodin	OH	CH_3_	H
3	Aloe–emodin	H	CH_2_OH	H
4	Rhein	H	COOH	H
5	Physcion	OCH_3_	CH_3_	H
6	Emodin 8-*O*-β-d-glucoside	OH	CH_3_	Glu

**Table:** **(b) Anthrones** 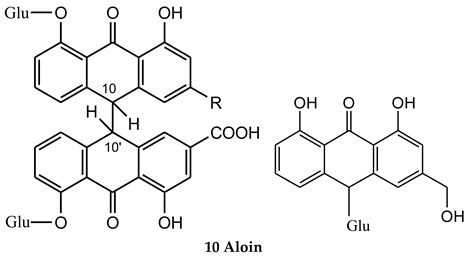

No.	Compound Name	R
7	Sennoside A (10,10′-trans)	COOH
8	Sennoside B (10,10′-meso)	COOH
9	Sennoside C (10,10′-trans)	CH_2_OH

**Table:** **(c) Stilbenes** 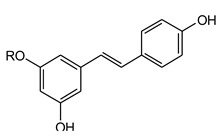

No.	Compound Name	R
11	Piceid	Glu
12	Resveratrol	H

**Table:** **(d) Flavonoids** 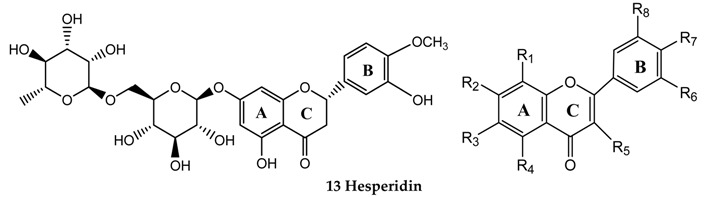

No.	Compound Name	R_1_	R_2_	R_3_	R_4_	R_5_	R_6_	R_7_	R_8_
14	Luteolin	H	OH	H	OH	H	H	OH	OH
15	Nobiletin	OCH_3_	OCH_3_	OCH_3_	OCH_3_	H	OCH_3_	OCH_3_	H
16	Sinensetin	H	OCH_3_	OCH_3_	OCH_3_	H	OCH_3_	OCH_3_	H
17	Tangeretin	OCH_3_	OCH_3_	OCH_3_	OCH_3_	H	H	OCH_3_	H
18	Vitexin	Glu	OH	H	OH	H	H	OH	H
19	Quercetin	H	OH	H	OH	OH	H	OH	OH
20	Hyperoside	H	OH	H	OH	OGlu	H	OH	OH

## Supplementary Materials

Supplementary materials can be accessed at: http://www.mdpi.com/1420-3049/21/5/631/s1.
