# Supplementary material for: Characterization of the Principal Constituents of Danning Tablets, a Chinese Formula Consisting of Seven Herbs, by an UPLC-DAD-MS/MS Approach
Source: Molecules. 2016 May 14;21(5):631. doi: 10.3390/molecules21050631 (PMC6273105; doi:10.3390/molecules21050631)
Supplement: Supplementary file 1 [file molecules-21-00631-s001.pdf]

Supplementary Materials: Characterization of the Principal Constituents in Danning Tablets, A Chinese Formula Consisting of Seven Herbs, by An UPLC-DAD-MS/MS Approach

Changsen Zhan, Aizhen Xiong, Danping Shen, Li Yang and Zhengtao Wang

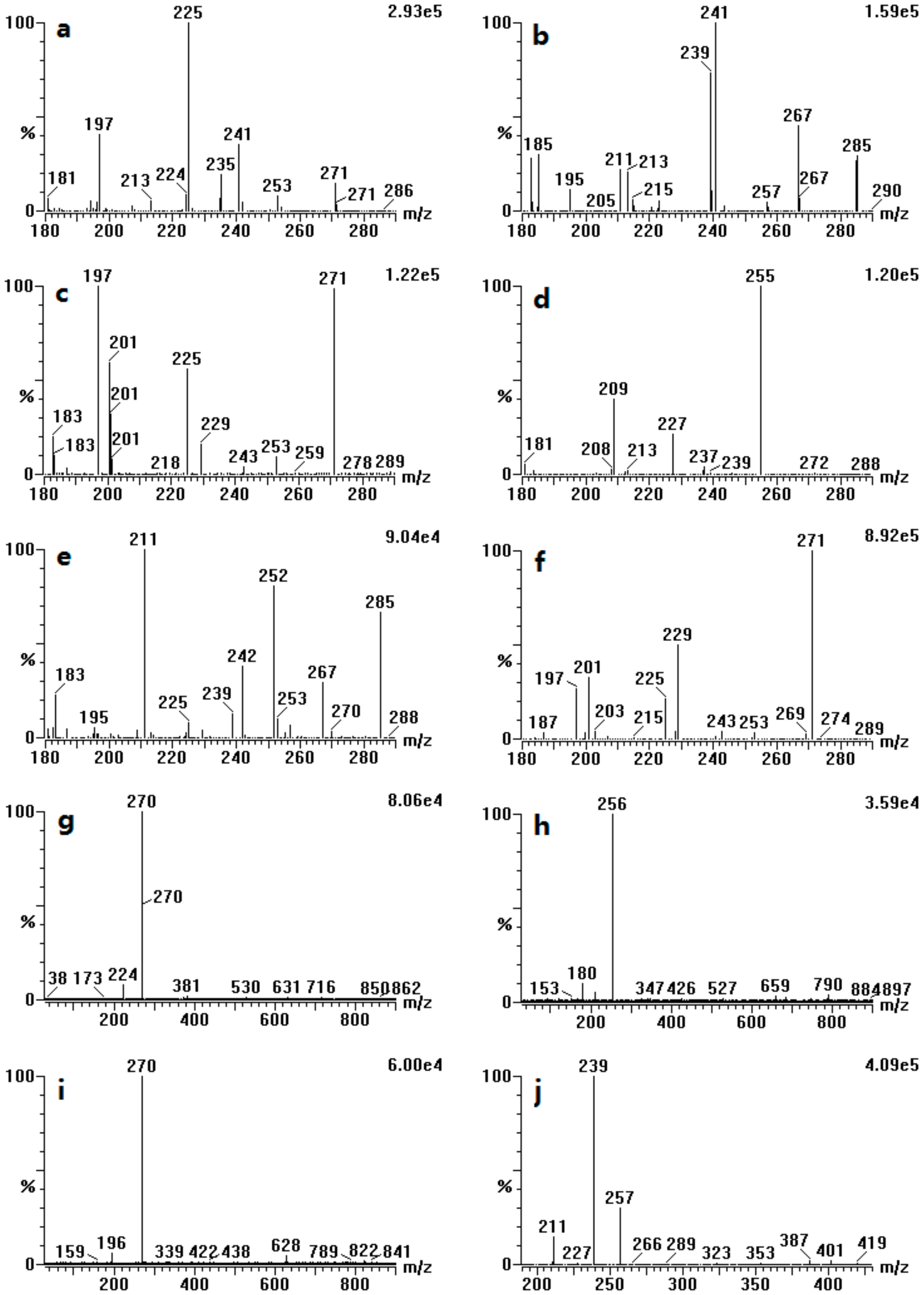

Figure S1. The MS/MS spectra for five free anthraquinones (**a**–**e**), 1 anthraquinone glycoside (**f**) and 4 anthrones (**g**–**j**) standard references (**a**. aloe-emodin; **b**. rhein; **c**. emodin; **d**. chrysophanol; **e**. physcion; **f**. emodin 8-*o*-β-D-glucoside; **g**. sennoside b; **h**. sennoside c ; **i**. sennoside a; **j**. aloin).

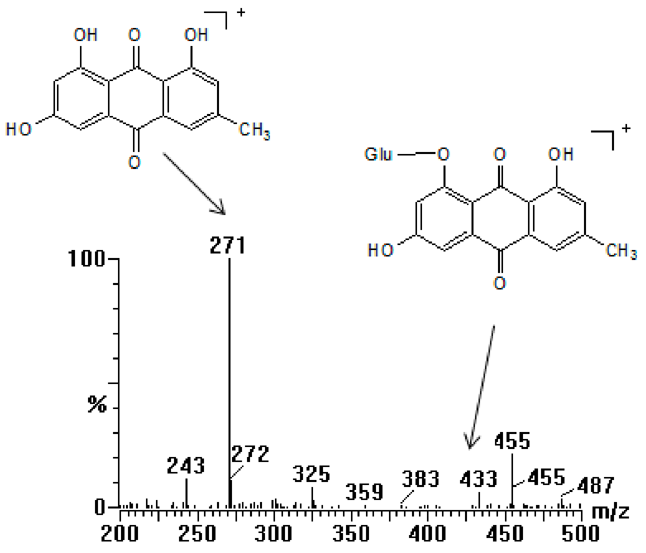

Figure S2. The full scan MS spectra for emodin 8-*O*-β-D-glucoside

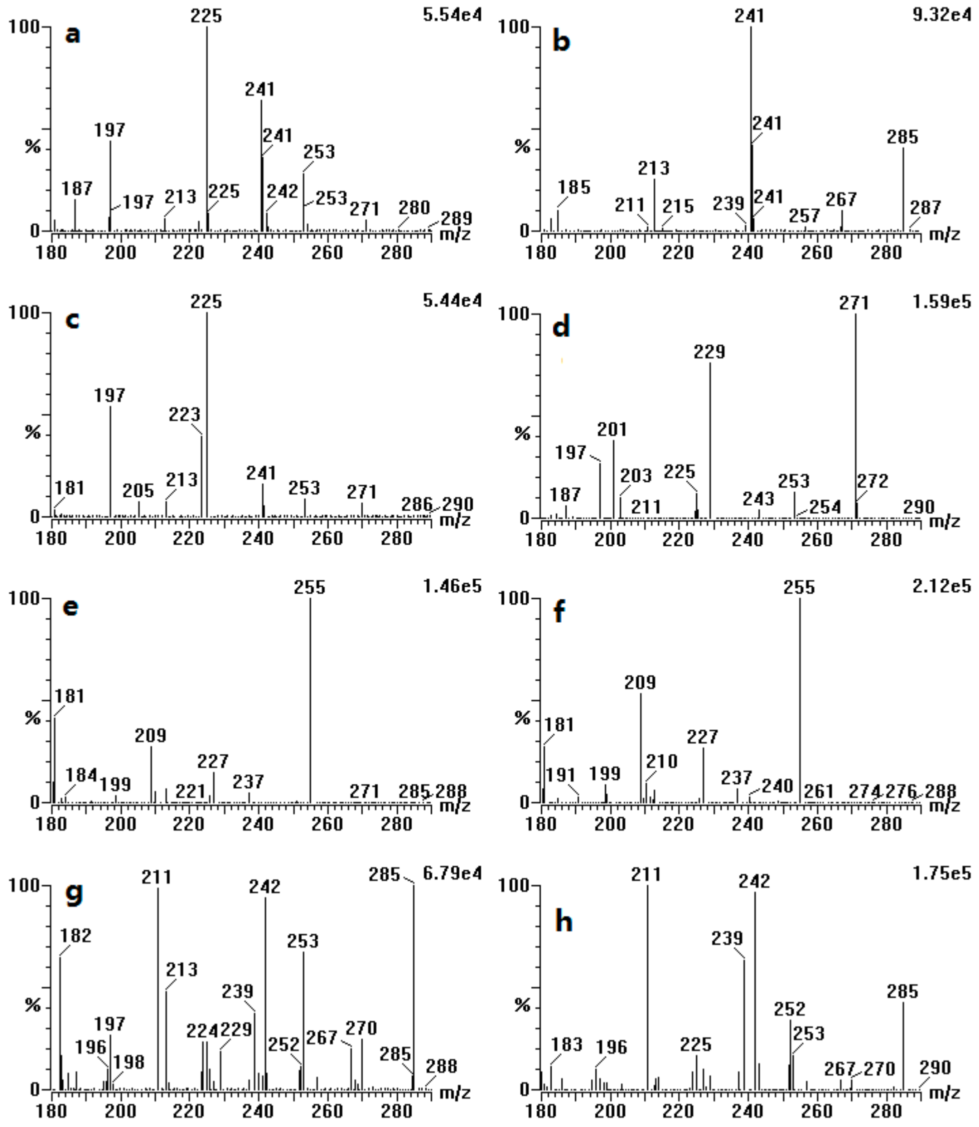

Figure S3. The MS/MS spectra for other anthraquinones (**a**. aloe-emodin-*O*-glucoside; **b**. rhein-*O*-glucoside; **c**. aloe-emodin-*O*-glucoside; **d**. emodin-*O*-glucoside; **e**. chrysophanol 1-*O*-glucoside; **f**. chrysophanol 8-*O*-glucoside; **g**. physcion 1-*O*-glucoside; **h**. physcion 8-*O*-glucoside).

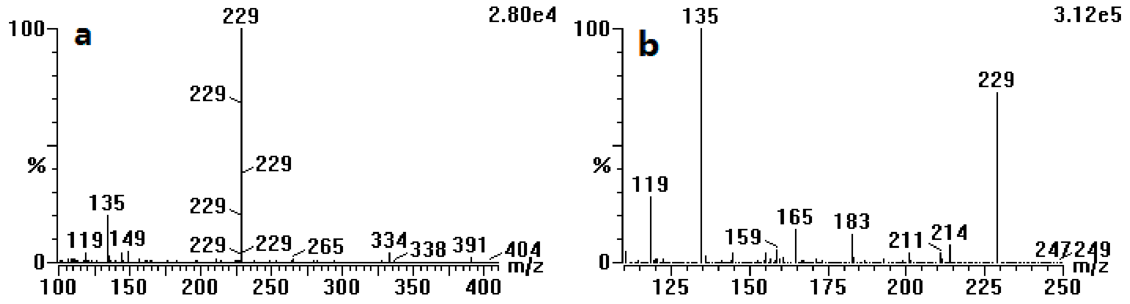

Figure S4. The MS/MS spectra for two stilbene standard references (**a**. piceid; **b**. resveratrol).

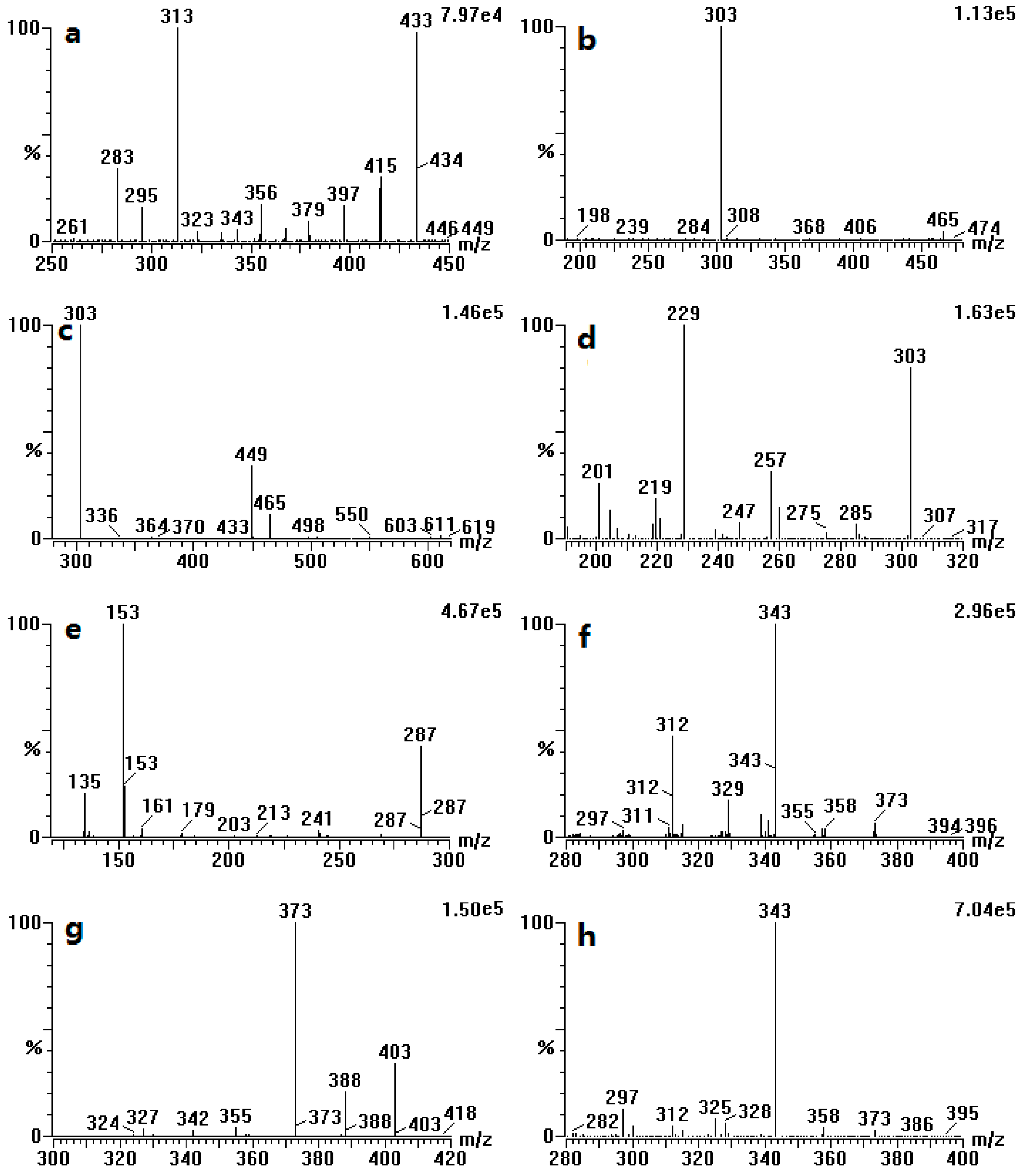

Figure S5. The MS/MS spectra for eight flavonoid standard references (**a**. vitexin; **b**. hyperoside; **c**. hesperidin; **d**. quercetin; **e**. luteolin; **f**. sinensetin; **g**. nobiletin; **h**. tangeretin).

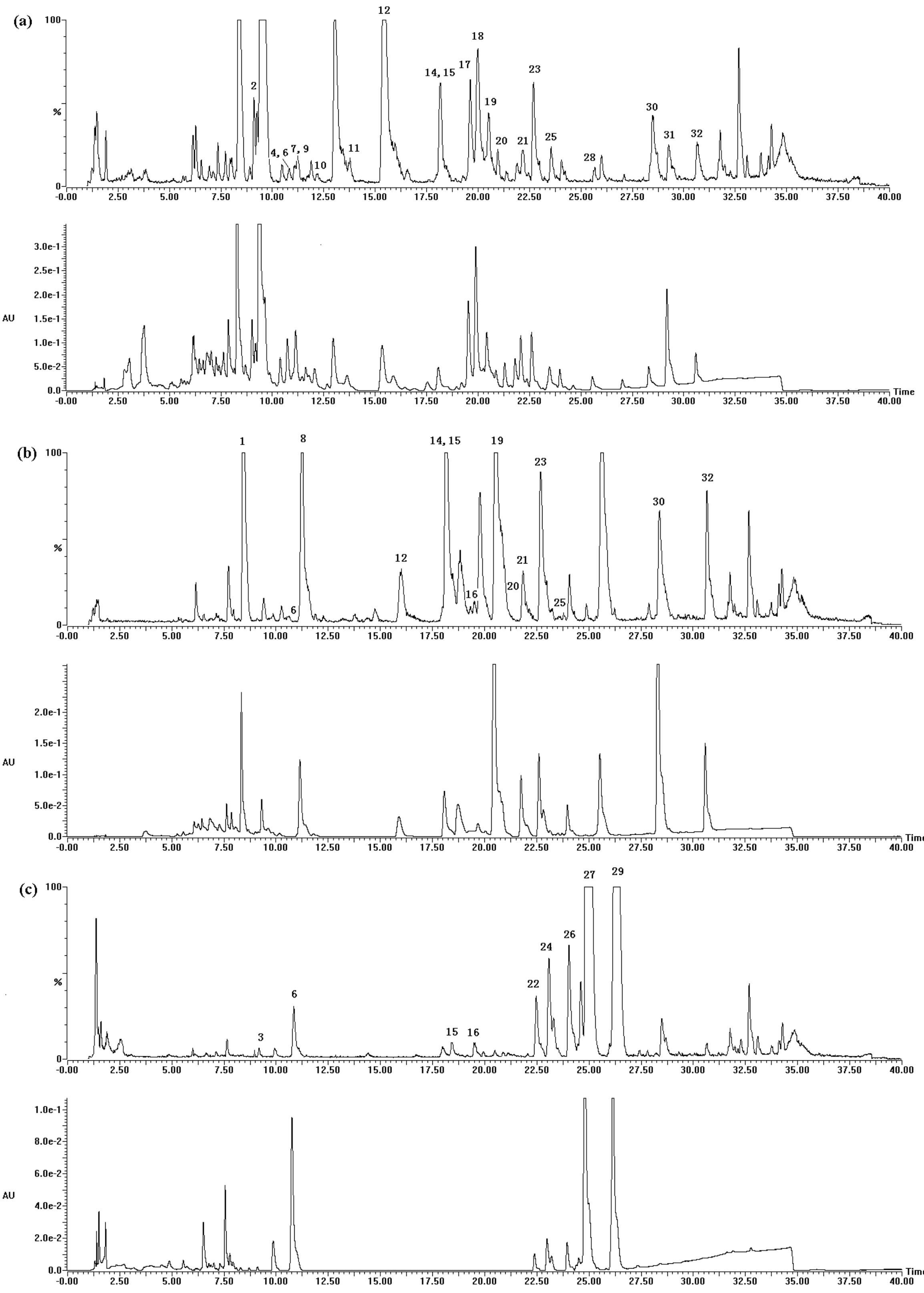

Figure S6. *Cont.*

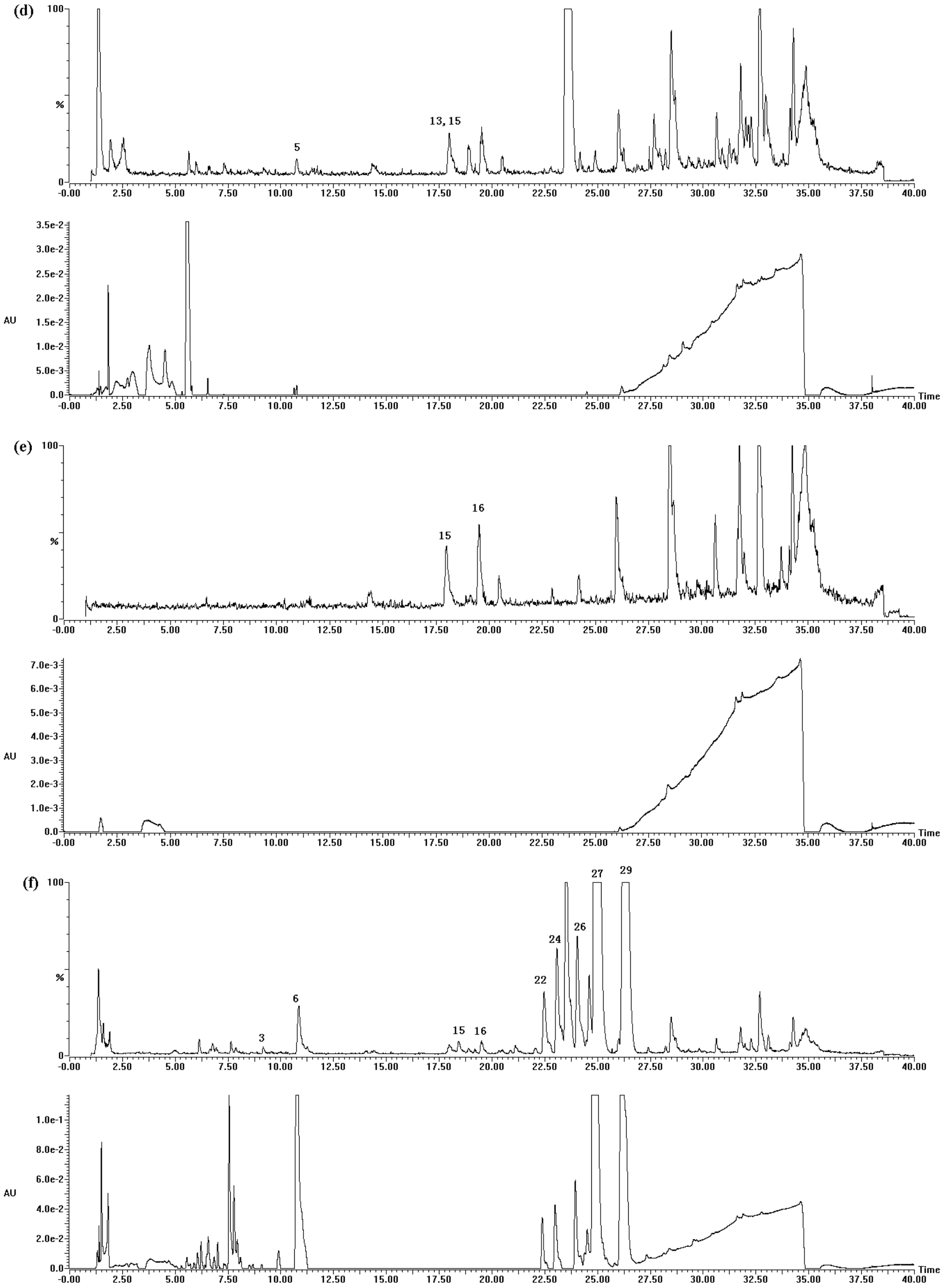

Figure S6. *Cont.*

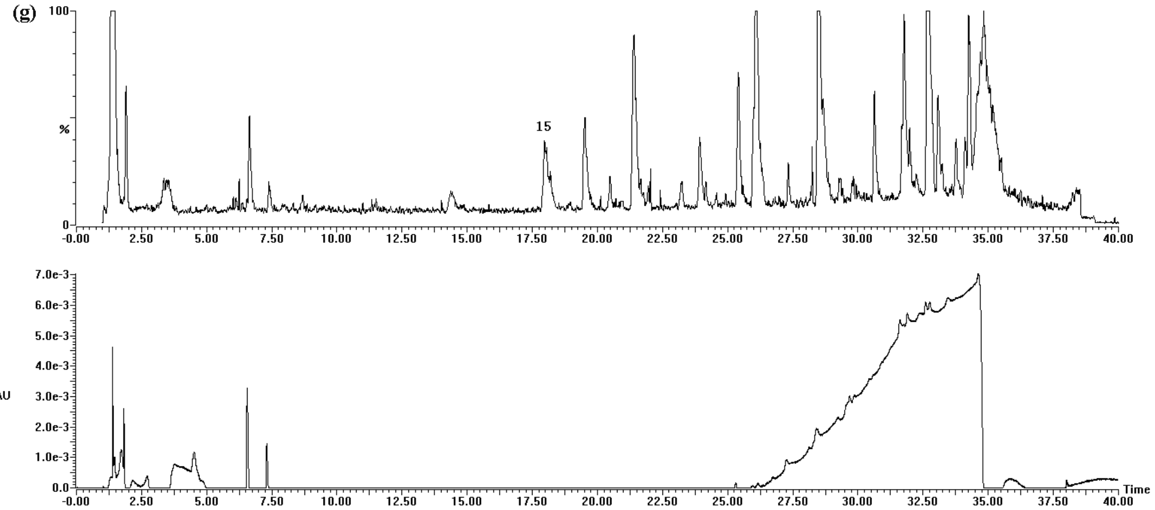

Figure S6. The UPLC-DAD-ESI-MS chromatograms of single herbs in Danning Tablets formula (**a**. *Rhei Radix et Rhizoma* (RRR); **b**. *Polygoni Cuspidati Rhizoma et Radix* (PCRR); **c**. *Citri Reticulatae Pericarpium* (CRP); **d**. *Citri Reticulatae Pericarpium Viride* (CRPV); **e**. *Curcumae Radix* (CR); **f**. *Crataegi Fructus* (CF); **g**. *Imperatae Rhizoma* (IR). Upper panel, LC-MS chromatogram at positive ion mode; lower panel, LC-UV chromatogram at 266 nm).

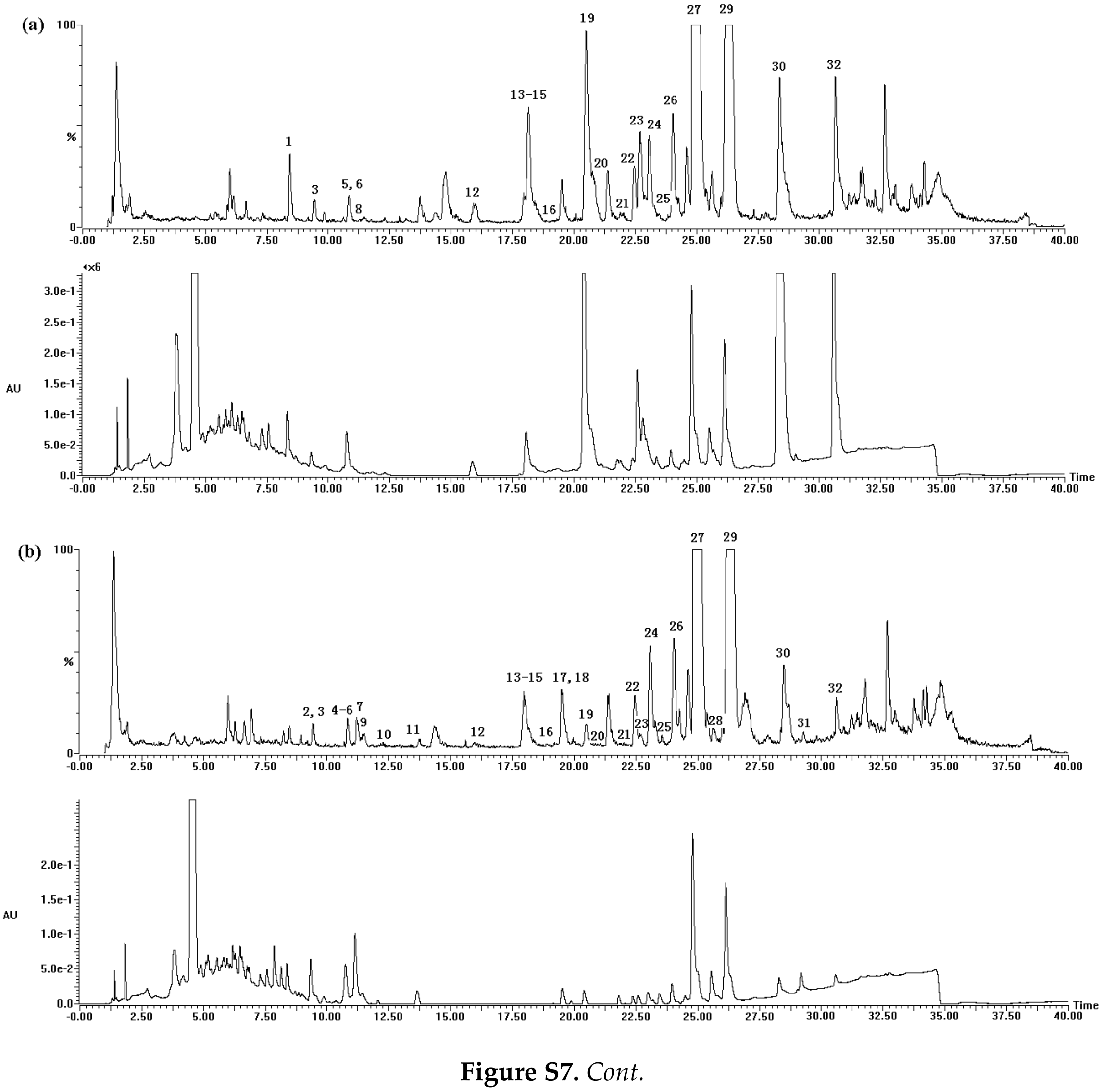

Figure S7. *Cont.*

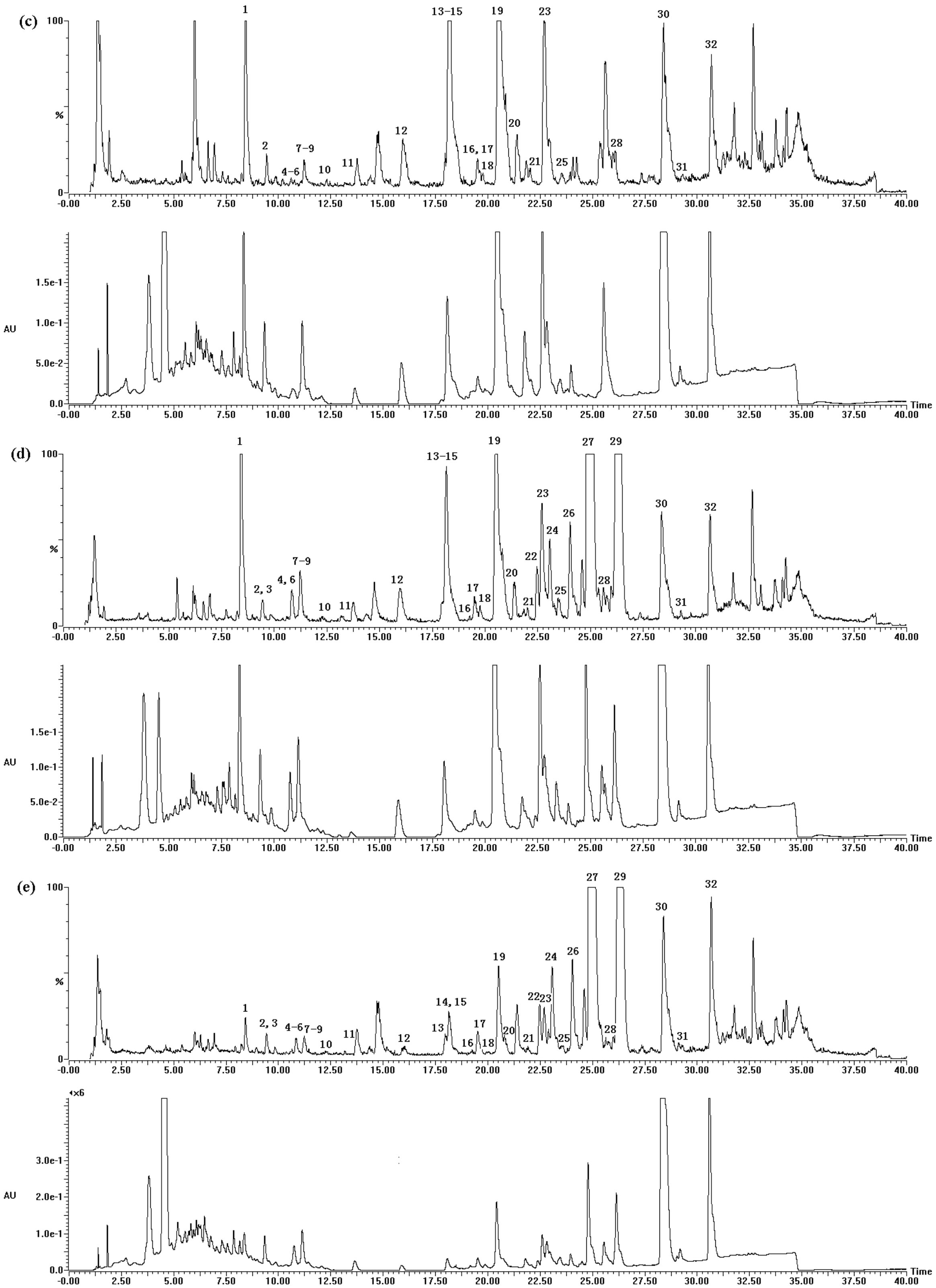

Figure S7. *Cont.*

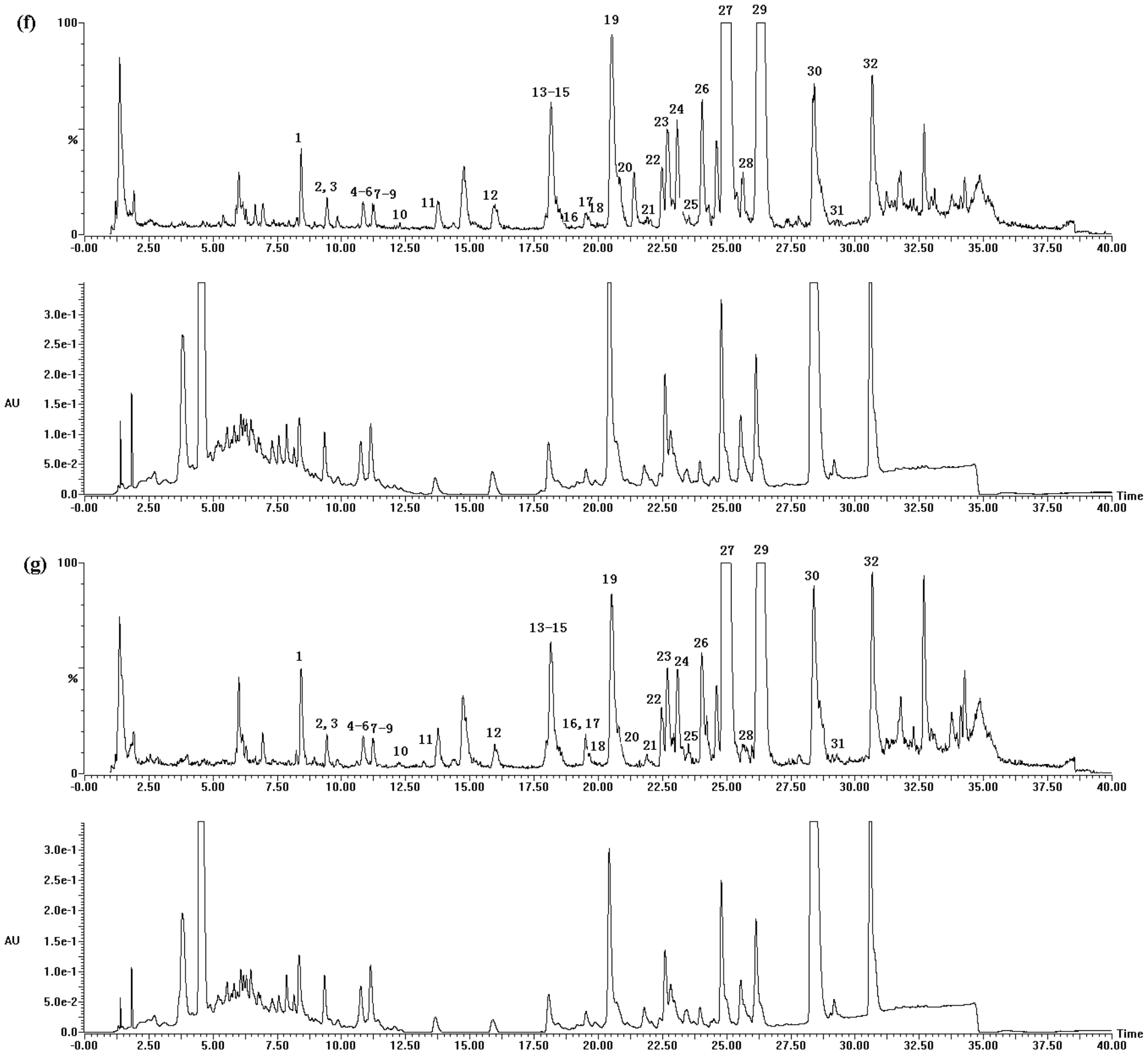

Figure S7. The UPLC-DAD-ESI-MS chromatograms of revised formulae of Danning Tablets with only six herbs (**a**. Revised formula without *Rhei Radix et Rhizoma* (RRR); **b**. Revised formula without *Polygoni Cuspidati Rhizoma et Radix* (PCRR); **c**. Revised formula without *Citri Reticulatae Pericarpium Viride* (CRPV); **d**. Revised fomula without Crataegi Fructus (CF); **e**. Revised fomula without Curcumae Radix (CR); **f**. Revised formula without *Citri Reticulatae Pericarpium* (CRP); **g**. Revised formula without *Imperatae Rhizoma* (IR). Upper panel, LC-MS chromatogram at positive ion mode; lower panel, LC-UV chromatogram at 266 nm).
